# Assessment on the coupling effects of drip irrigation and organic fertilization based on entropy weight coefficient model

**DOI:** 10.7717/peerj.3855

**Published:** 2017-10-03

**Authors:** Fenglin Zhong, Maomao Hou, Bizhu He, Iouzen Chen

**Affiliations:** 1 Department of Facility Agricultural Science and Engineering, College of Horticulture, Fujian Agriculture and Forestry University, Fuzhou, China; 2 Postdoctoral Research Center of Horticulture, College of Horticulture, Fujian Agriculture and Forestry University, Fuzhou, China; 3 Department of Horticulture, National Taiwan University, Taipei, Taiwan

**Keywords:** Organic fertilizer, Drip irrigation, Entropy weight, *Solanum lycopersicum* L, Yield, Water use efficiency

## Abstract

Water and fertilizer are two important factors influencing crop growth, development and yield formation. To investigate their combined effects on the soil-plant system, and to find out the optimal water and organic fertilizer coupling strategy for tomato (*Solanum lycopersicum* L), an experiment was carried out from May to October in 2016 in the south of China. The experiment consisted of three drip irrigation quotas (150, 180, 210 m^3^/ha) and three organic fertilizer application amounts (2,800, 3,600, 4,400 kg/ha). A water-fertilizer treatment (abbreviated as CK) that is in line with local practice was used for comparison. The tomato marketable yield, sugar/acid ratio (SAR) and irrigation water use efficiency (IWUE), as well as the soil salinity and available nutrient concentrations were measured. The results showed that the marketable yield was highly significantly (*p* < 0.01) affected by irrigation or fertilization. The SAR of tomato were significantly (*p* < 0.05) affected by irrigation or/and fertilization. The fertilization had an highly significant (*p* < 0.01) effect on the concentrations of soil nutrients (N, P, K), while the coupling effect of irrigation and fertilization was not pronounced. According to the multi-index analysis and the computed result by the entropy weight coefficient model, a 180 m^3^/ha irrigation quota in combination with 4,400 kg/ha organic fertilizer application amount was the optimal water-fertilizer coupling strategy which owned the most satisfactory comprehensive benefits. The marketable yield, SAR and IWUE under this optimal strategy were 122.4 t/ha, 9.2, 32.4 kg/m^3^, respectively, and by 28.0%, 29.6% and 28.1% higher compared to that under CK.

## Introduction

Drip irrigation can flexibly control the amount and position of soil moisture to meet crop requirements, which is proven to increase the crop water use efficiency and reduce the water loss (Xing et al., 2015). Presently, drip irrigation has been widely used in many crops (Eranki et al., 2017; Sinha, Buttar & Brar, 2017; Tian et al., 2017), including in tomato (Lu, Zhang & Liang, 2016). For greenhouse cultivated tomato, the plant water requirements are mainly derived from irrigation. When water stress occurs, the root quickly sends signal to the whole plant, and the plant makes the response through altering the structure and chemical components of root, the growth of aboveground part and the yield formation of tomato (Hou, Zhu & Jin, 2016b). Conversely, superfluous water supplies weaken the active oxygen metabolism ability of the plant, and aggravate lipid peroxidation, thus negatively affect fruit quality (Lin et al., 2004).

Another factor impacting crop quality and yield is fertilizer usage. In China, inorganic fertilizer has been used since 1980s because of its convenience (Liang et al., 2013). China was reported to account for 90% of the worldwide increase in fertilizer use since 1981 (Liu & Diamond, 2005). However, a number of studies have revealed that the overuse of inorganic fertilizer results in low fertilizer use efficiency (Badr, Abou-Hussein & El-Tohamy, 2016), soil salinization (Chang et al., 2013), as well as groundwater pollution (Hou et al., 2016a). Accordingly, using organic fertilizer are one of the crucial approaches to reach a compromise between crop production and environment conservation. Organic fertilizer contains various nutrients, microorganisms and enzymes, which is able to promote root nutrient uptake, reserve moisture and fertility, enhance soil buffering capacity, increase crop yield, improve crop quality and reduce soil salinity (Guo et al., 2016; Li et al., 2017a; Liu et al., 2016; Salehi, Tasdighi & Gholamhoseini, 2016). Correct use of organic fertilizer also benefits the soil carbon sequestration and lowers the CO_2_ discharge (Yuan et al., 2017). In recent years, straw and animal feces are increasingly being used as fertilizer due to the development of planting and breeding industry. In China, there are 4,950 million tons of organic fertilizer resources available that can provide 74 million tons of nitrogen (N), phosphorus (P_2_O_5_) and potassium (K_2_O) (Li & Li, 2012). However, due to the restriction in technology and policy, only a small part of organic resources are made into fertilizer; this intensifies the environmental risk such as that the unavailable straws were burned in the open air (Liu & Jin, 2010). In regards to the application amount of organic fertilizer, early results showed that the increase in fertilizer amount significantly increased the biomass of plant aboveground part, plant nutrient uptake and crop yield, while excessive application of fertilizer lowered the velocity of plant N accumulation and caused the yield decrease (Wang & Jia, 2012); one reason is that excessive organic fertilizers in soil produce quantities of base ions (Meng, 2008). Hence, it is of great importance to develop suitable strategies for organic fertilizer application.

A number of studies have investigated the effect of drip irrigation or organic fertilization on plant and soil (Müller, Ranquet Bouleau & Perona, 2016; Reyes-Cabrera et al., 2016; Wang & Jia, 2012). To our knowledge, however, few studies have described their combined effects on the soil-plant system. Moreover, the optimization of water-fertilizer schemes with comprehensive consideration of crop performance and soil properties, is seldom studied. Therefore, the objective of this study is: (1) to study the coupled effects of drip irrigation and organic fertilization on the tomato blossom-end rot (BER) incidence, marketable yield, fruit sugar to acid ratio (SAR), and irrigation water use efficiency (IWUE); (2) to observe the variation in soil available nutrients and salt profile distribution under different drip irrigation and organic fertilization treatments; (3) to find out the optimal water-fertilizer scheme by considering both the plant and soil indicators.

## Material and Method

### Experimental site

The experiments were carried out from May to October in 2016 at the Fruit Science and Technology Demonstrative Base of Yunxiao county (latitude 23°57′38″N, longitude 117°20′5″E) in Fujian province of China (the experiments were permitted by its legal representative named Zhang Zhixiong). The climate of the experimental field is subtropical maritime monsoon. The average temperature from 2005 to 2015 was 21.3 °C. The average maximum and minimum temperature were 28.2 and 13.4 °C, occurring in July and January, respectively. The annual precipitation is 1730.6 mm, and the frost-free duration is 347 days. The soil physicochemical properties in plough layer (measured on May 2, 2016) of the experimental field were shown in Table 1.

**Table 1:** The soil physicochemical properties in plough layer of the experimental field.

Indicators	Salt content (g/kg)	Organic matter content (%)	Total N content (g/kg)	Available P_2_O_5_ concentration (mg/kg)	Available K_2_O concentration (mg/kg)	Bulk density (g/cm^3^)	Field capacity(%)
Value	2.72	2.1	0.96	32.5	133.3	1.39	22.9

**Notes.**

The total N content was measured using the Kjeldahl method.

### Experimental design

The experiments were conducted in a plastic-covered greenhouse with span of 8 m and length of 30 m. The total experimental area was approximately 75 m^2^. Before transplant, soil ridges were established to create suitable condition for the plant. The width of each soil ridge was 60 cm, the length was 3 m, and the height was 5 cm. A distance of 20 cm was left between two adjacent ridges. Each soil ridge cultivated 2 lines of tomato plants, with a row-to-row spacing of 30 cm and plant-to-plant spacing of 40 cm. In total, there were 16 tomato plants in one ridge. These 16 tomatoes were considered to be one treatment. Each treatment was replicated three times. The treatments were arranged side-by-side in one line. Plastic film with 60 cm depth was buried into the adjacent area between different treatments, to prevent their interaction in irrigation or fertilization. The tomato variety “Xilan” was adopted as the plant material, their seedlings were transplanted on May 18. The weeding and pest control were conducted according to the conventional practice.

The experiment included three drip irrigation quotas (I1: 150 m^3^/ha, I2: 180 m^3^/ha, I3: 210 m^3^/ha), and three application amounts of bioorganic fertilizer (F1: 2,800 kg/ha, F2: 3,600 kg/ha, F3: 4,400 kg/ha). A conventional treatment (abbreviated as CK), applied with inorganic fertilizer (180 kg/ha N, 90 kg/ha P_2_O_5_, 54 kg/ha K_2_O) and drip irrigation (180 m^3^/ha), was used for comparison. Thus, there were 3 × 3 + 1 = 10 treatments. The drip irrigation system adopted mosaic column pipe with 8 mm inner diameter. The distance between adjacent emitters was 30 cm, the drip flow was 2 L/h, and the operating pressure was 0.3 MPa. The irrigation was conducted each 6 days from May 21. During the whole growth period of 130 days, the tomatoes were irrigated 21 times. The bioorganic fertilizer consisted of dry straw, bean dreg and swine manure (provided by Nanjing Institute of Vegetable and Flower Science, Jiangsu, China) and was fermented using EM (effective microorganism, produced by AiMuLe Company, Ltd, Jiangsu, China), and contained 5% N, 2.5% P_2_O_5_ and 1.5% K_2_O. The inorganic fertilizer was prepared by urea (46% N), calcium superphosphate (16% P_2_O_5_) and potassium sulphate (50% K_2_O). According to the local practice, the total amount of fertilizer was distributed as the ratio of basal fertilizer: first fruit cluster fertilizer: second fruit cluster fertilizer = 1:1:1. When using as basal fertilizer, the bioorganic fertilizer was mixed evenly with the surface soil.

### Measurement

(1) BER incidence (%) and marketable yield (t/ha) of tomato. At harvest stage, the mature tomatoes were collected in batches then weighed to calculate the total tomato yield. Meanwhile, the yield of tomato infected with BER was recorded. The BER incidence is the ratio of BER infected tomato yield to the total yield. The marketable yield is the difference between the total and BER infected yield (Zhai, Yang & Hou, 2015).

(2) SAR of tomato. The tomatoes in the first and third layer of the plant were used for the SAR determination. In each treatment, six tomatoes (three from first layer and three from the third) with similar appearance were collected from three randomly selected plants. The SAR was the average SAR value of the tomato for the two layers.

The total sugar content was measured using the Fehling reagent titration method. The total acid content was measured using the sodium hydroxide titration method (Hou & Shao, 2016). The SAR is calculated as the ratio of total sugar to total acid content.

(3) IWUE (kg/m^3^) of tomato. Tomato IWUE is the ratio of marketable yield to the total irrigation amount (Shao et al., 2015). The total irrigation amount was calculated by the irrigation quota and irrigation times.

(4) Salt content in soil profile (g/kg). At seven days after the last irrigation (Sep 25), the soil samples in profile were collected using 5-point method as different soil layers of 0–20, 20–40 and 40–60 cm (Hou & Shao, 2016). The 5 points were in a line that was perpendicular to the tomato row, including the edge of the soil ridge (two points), the dripper position (two points), and the middle of two drippers (one point). The samples were naturally dried then grinded to measure the soil salt content (Chu, Kang & Wan, 2015). Average soil salt content from the 5 points was used for the following analysis.

(5) Soil available nutrient concentrations (mg/kg). The soil samples in 0–20 cm soil layer were air dried then passed through 2 mm sieve to determine soil available nutrient concentrations. The available N concentration was measured using the alkali solution diffusion method; the available P concentration was measured using the sodium bicarbonate extraction method; the available K concentration was measured using the ammonium acetate extraction method (Wei et al., 2017).

### Entropy weight coefficient model

The entropy weight coefficient model, which had been widely used in the decision of agricultural strategy (Hou, Shao & Zhai, 2014; Shao et al., 2012), was employed to select the optimal irrigation and fertilization scheme in this study. The modeling method is as follows.

Supposing that there are *n* evaluation indexes and *m* water-fertilizer schemes, *m* schemes in corresponding with *n* indexes can form a matrix:

\begin{eqnarray*}R={ \left( {r}_{ij} \right) }_{m\times n} \end{eqnarray*}

Where; *r*_*ij*_ is the *j*th evaluation index of the *i*th scheme. For *r*_*j*_, there is information entropy (average amount of information after excluding redundancy):

\begin{eqnarray*}{E}_{j}=-\sum _{i=1}^{m}{p}_{ij}~\ln \nolimits {p}_{ij}, \left( j=1,2,3,\ldots n \right) \end{eqnarray*}

And *p*_*ij*_ is calculated from the formula:

\begin{eqnarray*}{p}_{ij}={r}_{ij}/\sum _{i-1}^{m}{r}_{ij} \end{eqnarray*}

The entropy value of *j*th index:

\begin{eqnarray*}{e}_{j}= \frac{1}{\ln \nolimits m} {E}_{j},   \left( j=1, 2, 3,\ldots , n \right) \end{eqnarray*}

The objective weight of *j*th index:

\begin{eqnarray*}{\theta }_{j}= \left( 1-{e}_{j} \right) /\sum _{i=1}^{n} \left( 1-{e}_{j} \right) ,   \left( j=1, 2, 3, \ldots , n \right) \end{eqnarray*}

It is clear that:

\begin{eqnarray*}0\leq {\theta }_{j}\leq 1;\sum _{j=1}^{n}{\theta }_{j}=1 \end{eqnarray*}

The comprehensive index weight can be obtained by combining the subjective weight *w*_1_, *w*_2_, *w*_3_…*w*_*n*_ by decision maker with the objective weight *θ*_*j*_ ( *j* = 1, 2, 3, …, *n*):

\begin{eqnarray*}{\alpha }_{j}={\theta }_{j}\overline{{\omega }_{j}}/\sum _{j=1}^{n}{\theta }_{j}\overline{{\omega }_{j}},   \left( j=1, 2, 3,\ldots , n \right) \end{eqnarray*}

The optimum value of each row was recorded as *r*_j_*, and the index value in the matrix (*R*) were normalized. The indexes can be divided into two classes, namely the profitable index and the damnous index. For profitable index (marketable yield, SAR, IWUE, available N, P and K), the index value is expected to be higher. While for damage index (soil salinity), the index value is expected to be lower. The normalization methods for the profitable and damage index were:

\begin{eqnarray*}{d}_{ij}= \left\{ \begin{array}{@{}c@{}} \displaystyle \frac{{r}_{ij}}{{r}_{j}^{\ast }} ,    {r}_{j}^{\ast }=\max \left\{ {r}_{ij} \right\} \\ \displaystyle \frac{{r}_{j}^{\ast }}{{r}_{ij}} ,    {r}_{j}^{\ast }=\min \left\{ {r}_{ij} \right\} \end{array} \right. \end{eqnarray*}

The entropy weight evaluation value (the optimal scheme will obtain the highest entropy weight evaluation value) of each water-fertilizer scheme can be calculated from:

\begin{eqnarray*}{\lambda }_{i}=\sum _{j=1}^{n}\alpha {d}_{ij},  i=1, 2, 3,\ldots , m. \end{eqnarray*}

### Statistic analysis

Data were submitted to SPSS 18.0 software to compare the difference (Hou et al., 2017b).

## Result

### The effect of different water-fertilizers on tomato BER incidence and marketable yield

**Figure 1:**
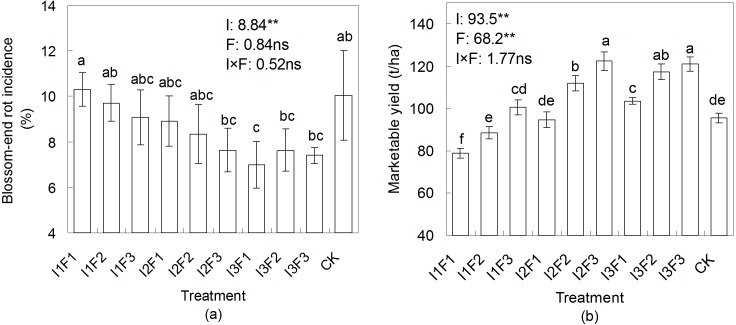
The effect of different water-fertilizer treatments on the blossom-end rot incidence and the marketable yield of tomato. I1, I2 and I3 represent different irrigation quotas of 150, 180 and 210 m^3^/ha. F1, F2 and F3 represent different organic fertilizer application amounts of 2,800, 3,600 and 4,400 kg/ha. Means followed by the same letter (a, b, c, d, e, or f) do not differ significantly at 0.05 level, according to Duncan’s multiple range test (Lawrence, 1984). **and ns indicate that the experimental treatment has an highly significant (at 0.01 level) effect and no significant effect on blossom-end rot incidence or marketable yield, respectively. The figures prior to **and ns were the *F* values.

The BER incidence and marketable yield of tomato for different treatments are shown in Fig. 1. The tomato BER incidence was significantly (*p* < 0.01) affected by irrigation. The highest BER incidence of 10.3% was found in I1F1, whereas the lowest (7.0%) was in I3F1. Overall, the BER incidence decreased as the irrigation quota or the fertilizer application amount increased.

Irrigation or fertilization had significant (*p* < 0.01) effect on the marketable yield of tomato (Fig. 1). The tomato marketable yield was generally increased when the irrigation quota or the fertilizer application amount increased. However, the marketable yield was not significantly (*P* > 0.05) affected by the combination of irrigation and fertilization. The highest marketable yield of tomato, 122.4 t/ha, was obtained by I2F3, which was significantly (*p* < 0.05) higher when compared to most other treatments, and was 28% higher than that by CK (95.6 t/ha).

### The effect of different water-fertilizers on tomato SAR

**Figure 2:**
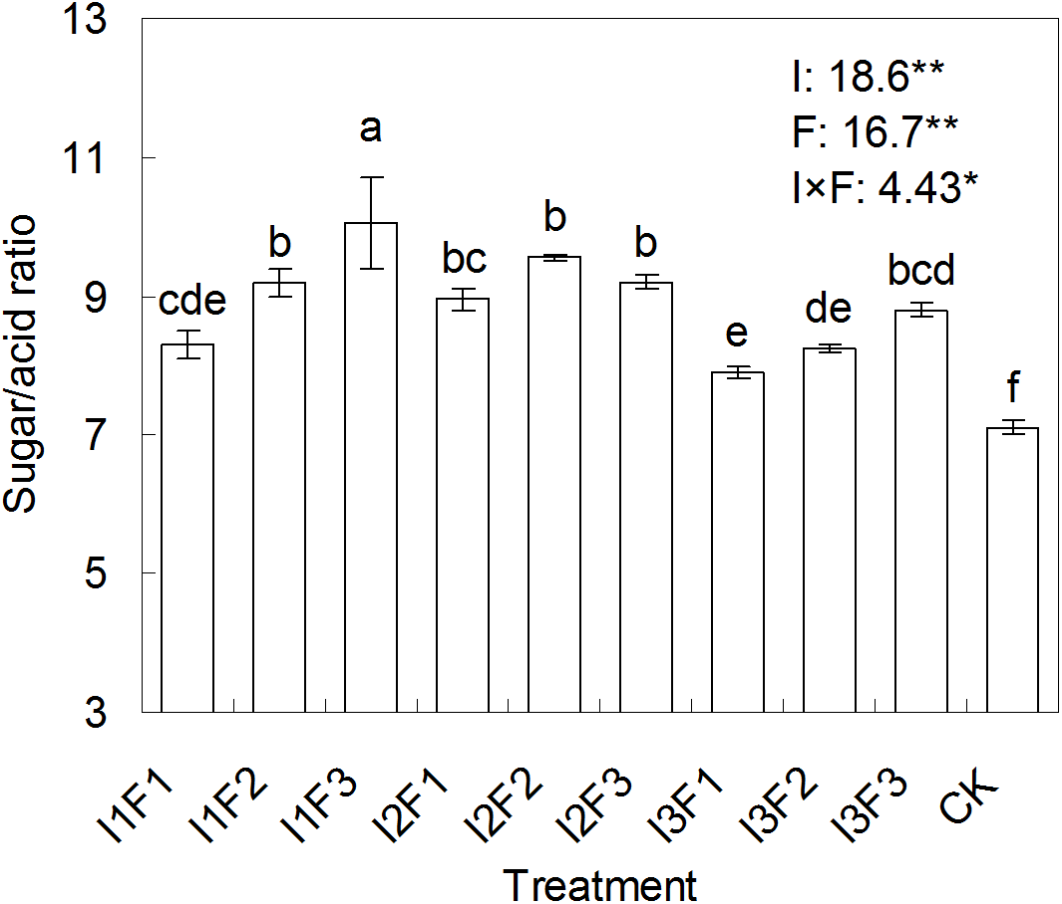
The effect of different water-fertilizer treatments on the sugar/acid ratio of tomato. I1, I2 and I3 represent different irrigation quotas of 150, 180 and 210 m^3^/ha. F1, F2 and F3 represent different organic fertilizer application amounts of 2,800, 3,600 and 4,400 kg/ha. In the same column, means followed by the same letter (a, b, c, d, e, or f) do not differ significantly at 0.05 level, according to Duncan’s multiple range test (Lawrence, 1984). * and ** indicate that the experimental treatment has a significant (at 0.05 level) effect and an highly significant (at 0.01 level) effect on the tomato sugar/acid ratio, respectively. The figures prior to **and ns were the *F* values.

Figure 2 gave the SAR of tomato fruit under different water-fertilizer treatments. The tomato SAR was significantly (*p* < 0.01) affected by irrigation or fertilization, and was also significantly (*p* < 0.05) affected by their combination. The SAR of tomato under the different treatments were 11.2–41.5% higher than that under CK. The highest SAR of 10.1% was registered by I1F3, and was significantly (*p* < 0.05) higher in comparison to other treatments, while the lowest (7.9%) was found in I3F1. The result indicated that the irrigation quota of 150 m^3^/ha combined with organic fertilizer application amount of 4,400 kg/ha was most conductive to achieving a high SAR in the tomato fruit.

### The effect of different water-fertilizers on the tomato IWUE

**Figure 3:**
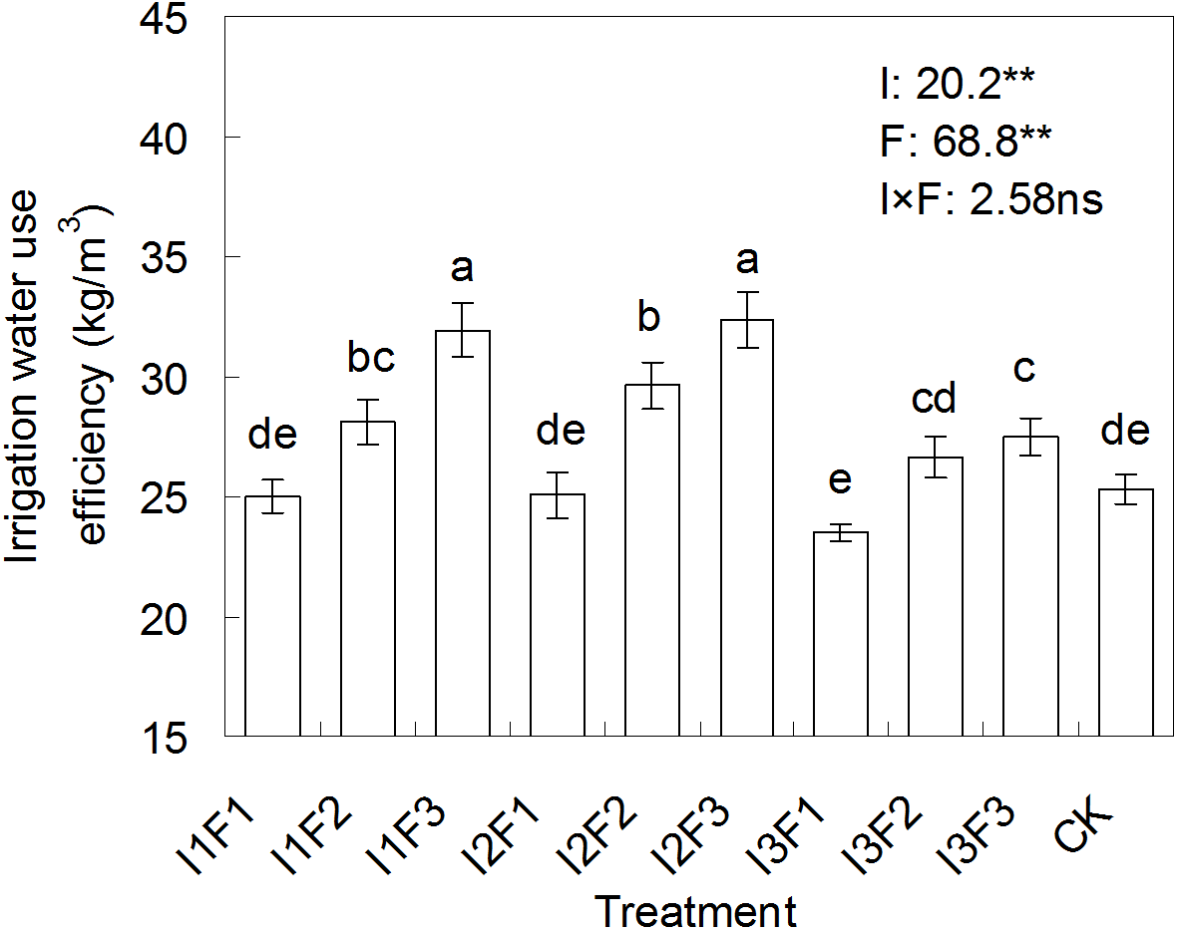
The effect of different water-fertilizer treatments on the irrigation water use efficiency of tomato. I1, I2 and I3 represent different irrigation quotas of 150, 180 and 210 m^3^/ha. F1, F2 and F3 represent different organic fertilizer application amounts of 2,800, 3,600 and 4,400 kg/ha. Means followed by the same letter (a, b, c, d, or e) do not differ significantly at 0.05 level, according to Duncan’s multiple range test (Lawrence, 1984). ** and ns indicate that the experimental treatment has a highly significant (at 0.01 level) effect and no significant effect on the irrigation water use efficiency. The figures prior to **and ns were the *F* values.

The tomato IWUE for different treatments are displayed in Fig. 3. The IWUE of tomato was significantly (*p* < 0.01) affected by irrigation or fertilization. However, IWUE was insignificantly (*P* > 0.05) affected by the combination of irrigation and fertilization. The highest IWUE was detected for I2F3, and was 28.1% higher relative to CK.

### The effect of different water-fertilizers on salt distribution in soil profile

**Figure 4:**
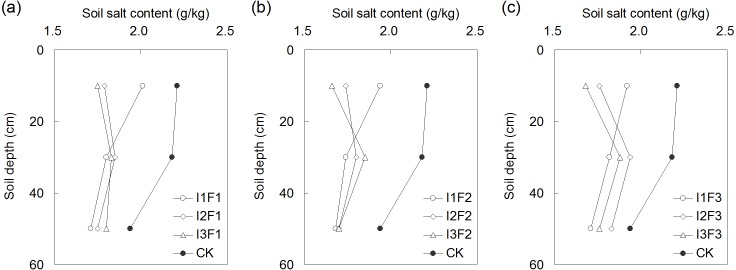
The salt distribution in soil profile under the different organic fertilizer application amounts of F1 (A), F2 (B) and F3 (C). I1, I2 and I3 represent different irrigation quotas of 150, 180 and 210 m^3^/ha. F1, F2 and F3 represent different organic fertilizer application amounts of 2,800, 3,600 and 4,400 kg/ha.

The treatments reduced soil salinity in all soil layers compared to CK (Fig. 4). The lowest salt content in the plough layer was observed in I3F2, and was 1.66 g/kg. Under the same organic fertilizer application amount, the salt content in plough layer decreased as the irrigation quota increased. However, the increase in irrigation quota resulted in a higher salt content in 20–40 cm soil layer, this was particularly obvious when the organic fertilizer application amount was 3,600 kg/ha (Fig. 4B). From the perspective of the total salt content in 0–60 cm soil layer, I3F2 had the lowest soil salt content which was 17.7% lower compared to CK.

### The effect of different water-fertilizers on soil available nutrient concentrations

The soil available nutrient concentrations under the different treatments are shown in Table 2. After one-season cultivation of tomato, the concentrations of available N, P and K were in ranges of 140.5–172.8 mg/kg, 17.1–20.1 mg/kg and 118.0–141.3 mg/kg, respectively. The highest concentrations of available N and K were both registered by I2F3, while the highest available P concentration was in I3F3. Relative to CK, I1F1, I1F2 and I3F1 significantly (*p* < 0.05) decreased soil available N concentration; I1F1, I2F1, I2F2 and I3F1 significantly (*p* < 0.05) decreased soil available *P* concentration; while, no treatments had significant (*P* > 0.05) effect on the concentration of soil available K. It should be noticed that, since the soil available P concentrations under the treatments were all lower compared to that under CK, addition of a P supplement may need to be considered when using the organic fertilizer of this study.

**Table 2:** The effect of different water-fertilizer treatments on soil available nutrient concentrations.

Treatment	Available N (mg/kg)	Available P (mg/kg)	Available K (mg/kg)
I1F1	140.5 ± 5.87^e^	17.1 ± 0.26^cd^	118.0 ± 2.81^c^
I1F2	144.4 ± 6.67^de^	18.2 ± 0.86^abcd^	129.7 ± 7.57^abc^
I1F3	168.7 ± 5.55^ab^	19.1 ± 1.23^abc^	130.0 ± 4.05^abc^
I2F1	153.4 ± 5.66^cde^	16.7 ± 0.57^d^	126.0 ± 7.05^abc^
I2F2	157.3 ± 2.99^bcd^	17.5 ± 0.68^bcd^	133.2 ± 6.65^abc^
I2F3	172.8 ± 5.96^a^	19.7 ± 1.64^ab^	141.3 ± 6.83^a^
I3F1	147.2 ± 7.49^de^	17.5 ± 0.93^bcd^	124.4 ± 4.91^bc^
I3F2	153.8 ± 5.95^cde^	18.2 ± 0.69^abcd^	130.8 ± 7.62^abc^
I3F3	158.9 ± 9.51^abcd^	20.1 ± 1.09^a^	137.0 ± 6.23^ab^
CK	163.6 ± 6.61^abc^	20.2 ± 1.59^a^	130.8 ± 8.70^abc^
I	250^*^	0.68ns	2.34ns
F	955^**^	10.7^**^	7.12^**^
I × F	81.4ns	0.24ns	0.24ns

**Notes.**

I1, I2 and I3 represent the different irrigation quotas of 150, 180 and 210 m^3^/ha. F1, F2 and F3 represent the different organic fertilizer application amounts of 2,800, 3,600 and 4,400 kg/ha. In the same column, means followed by the same letter (a, b, c, d, or e) do not differ significantly at 0.05 level, according to Duncan’s multiple range test. *, **and ns indicate that the experimental treatment has a significant (at 0.05 level) effect, a highly significant (at 0.01 level) effect, and no significant effect on soil available nutrient concentrations (N, P, K), respectively. The figures prior to **and ns were the *F* values.

### Entropy weight coefficient assessment on the different water-fertilizer schemes

**Figure 5:**
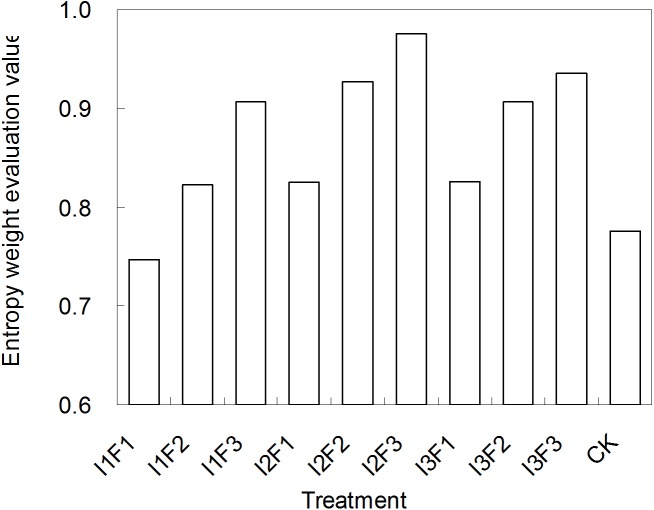
The entropy weight evaluation values for the different water-fertilizer treatments. I1, I2 and I3 represent different irrigation quotas of 150, 180 and 210 m^3^/ha. F1, F2 and F3 represent different organic fertilizer application amounts of 2,800, 3,600 and 4,400 kg/ha. The treatment with higher entropy weight evaluation value has a better comprehensive effect including yield increase, quality improvement, soil salinity reduction, etc.

Seven indicators including marketable yield, SAR, IWUE, soil salt content (in plough layer), soil available N, P and K concentrations, were comprehensively considered in order to select the optimal irrigation and fertilization coupling scheme. Based on the principle of entropy weight coefficient model, the seven indicators were all classified as the profitable index except “soil salt content”. To avoid human impact, the subjective weights of these seven indicators were assigned in sequence as 0.200, 0.200, 0.200, 0.200, 0.067, 0.067, 0.067. The calculated objective weights were 0.325, 0.159, 0.192, 0.137, 0.071, 0.075, 0.040. Thus, the comprehensive weights were 0.371, 0.182, 0.220, 0.156, 0.027, 0.029, 0.015, in order. The entropy weight evaluation values for the schemes were shown in Fig. 5. As mentioned above, the scheme with a higher entropy weight evaluation value had a better comprehensive benefit including the yield increase, quality improvement, soil salinity reduction, etc., therefore, I2F3 was the optimal scheme which obtained the highest entropy weight evaluation value of 0.975, followed by I3F3, while I1F1 was most inefficient.

**Figure 6:**
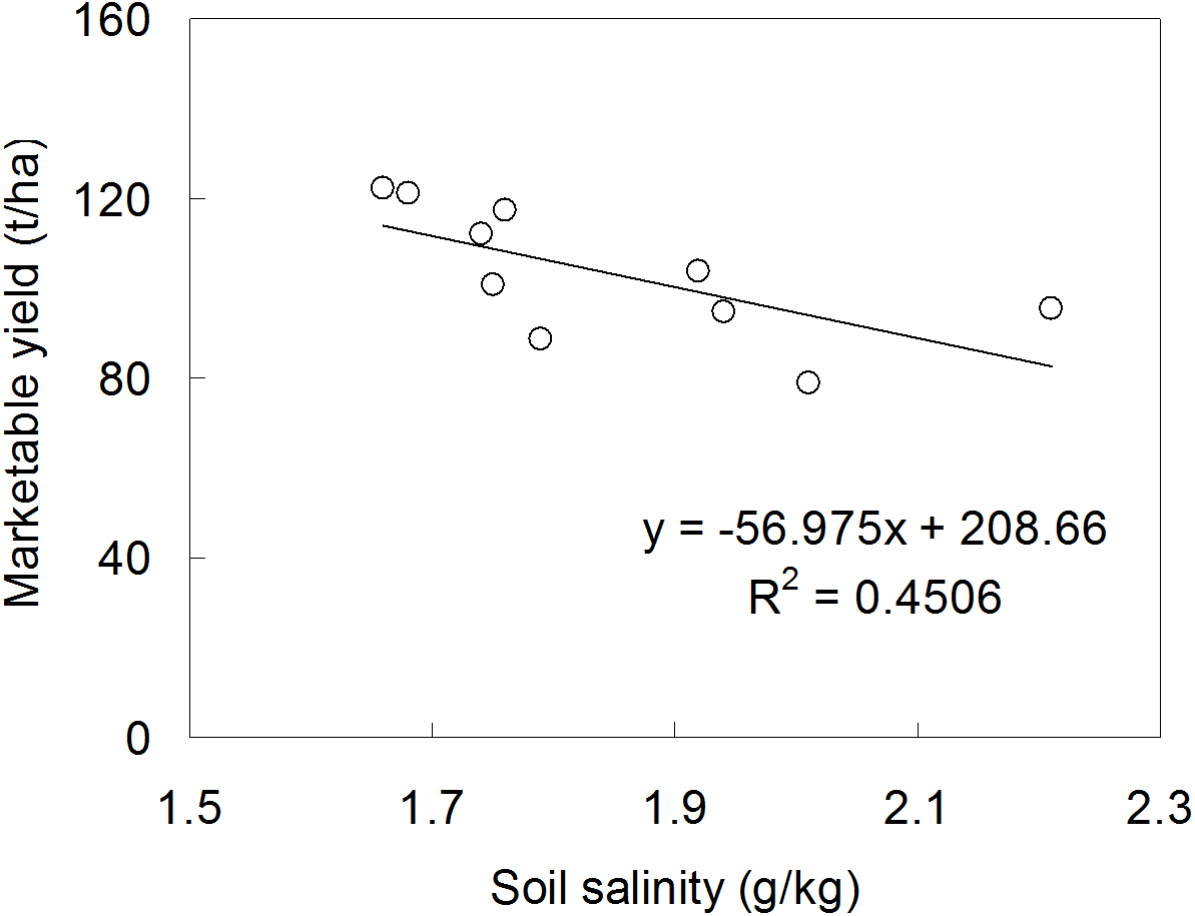
The relationship between soil salinity and marketable tomato yield.

## Discussion

Our study detected a decrease in BER incidence with increased irrigation quota, which confirmed the early finding by Adams & Ho (1993). Although soil moisture is not the root cause of BER, the tomato BER is exacerbated when an insufficient amount of water is applied (Pill, 1980). The reason might be that insufficient water supply hindered the plants ability to obtain nutrients, particularly the calcium, to move to the end of tomato fruit (Zhai, Yang & Hou, 2015). Moreover, in our case, the increase in the organic fertilizer amount generally lowered the BER incidence. There might be two reasons: (1) organic fertilizer provided more complete nutrients including trace elements which guaranteed the healthy growth of plant (Zhang et al., 2012); (2) salt stress was proved to be another important inducement of tomato BER (Okano, 2002), the organic acid released by organic fertilizer was able to chelate the soil salt ions, therefore alleviating the plant damage caused by the soil salinity and reduced the BER incidence.

The previous study by Jenkison (1994) noted that, due to the continuous nutrient mineralization and the residual effect cumulation, organic fertilizer was more advantageous in the long-term manurial effect compared to inorganic fertilizer. While, in our experiment, the organic fertilizer treatment (I2F3) achieved a significantly higher yield in only one season (Fig. 1). This might relate to the soil characters in our experimental field. The field was applied with inorganic fertilizer in a long duration which resulted in a high soil salinity (2.72 g/kg) before our experiment. The yield increase under the organic fertilization was more likely due to the reduced soil salinity in plough layer (Fig. 6), while not due to the nutrients provided by the fertilizer, since the measured available nutrient concentrations in soil under organic fertilizer treatments were similar compared to CK (Table 1). Besides, the yield under treatments I3F2 and I3F3 were not significantly different from treatment I2F3, indicating that there might be more than one suitable water-fertilizer scheme for tomato. Moreover, the increase in irrigation quota (I3F3) upon the optimal scheme (I2F3) was invalid in increasing the tomato yield.

**Figure 7:**
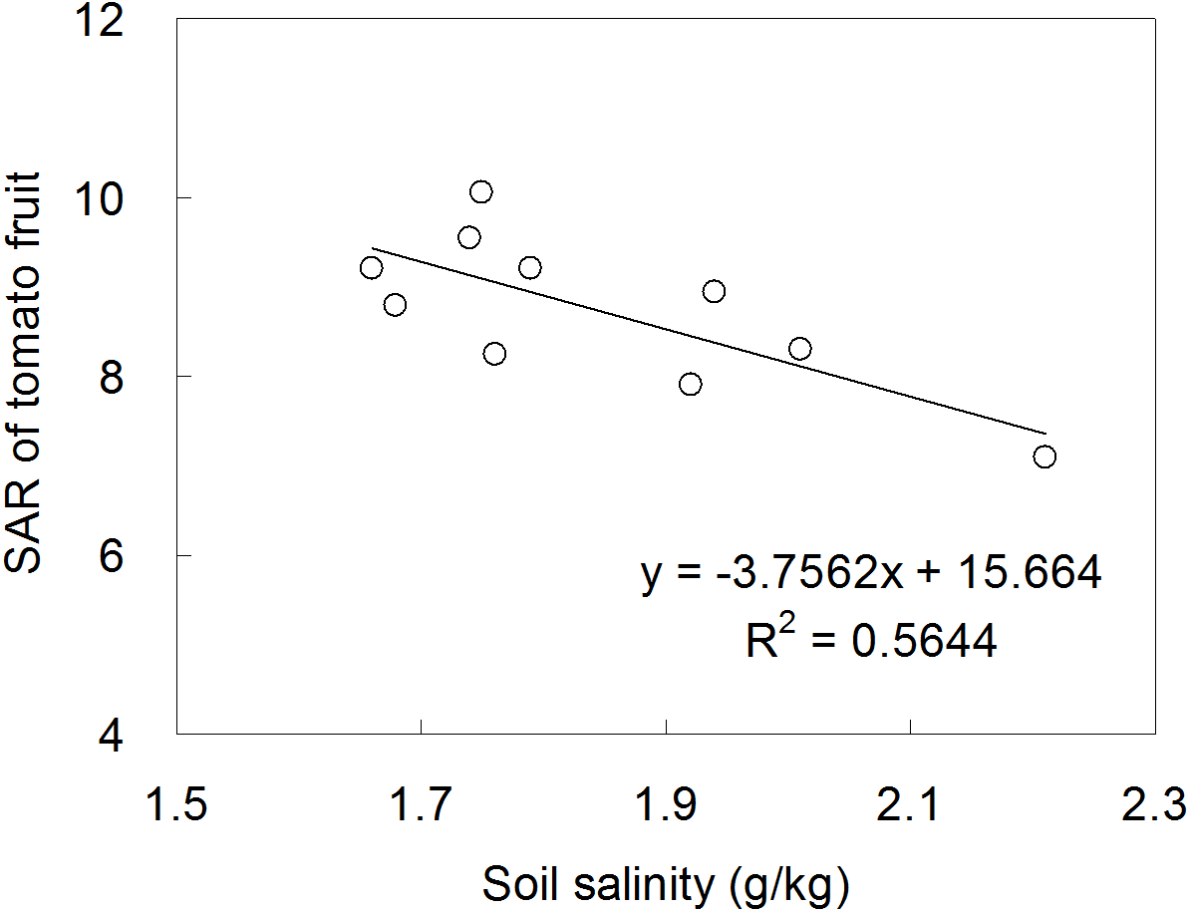
The relationship between soil salinity and tomato SAR.

SAR is one of the indicators that is used to characterize the fruit quality and taste (Wu et al., 2014). Our study found a significant coupling effect of irrigation and fertilization on SAR, which was in line with Xing et al.’s (2015) result. Interestingly, greatest SAR was detected in I1F3, in which treatment the tomato was applied with the lowest irrigation quota but the highest organic fertilizer amount. The early studies gave the reasons: (1) the decrease in irrigation amount reduced the water consumption used for osmotic regulation in plant, and increased the content of sugar that entering from phloem to fruit (Hou et al., 2017a); (2) the increase in organic fertilizer application amount might have resulted in an increase in the plant uptake of microelements, particularly those greatly contributed to fruit quality such as zinc and potassium (Angeles Botella et al., 2017; Li et al., 2017b). Otherwise, the increase in SAR under organic fertilization possibly correlated to the decrease in salinity in plough layer (Fig. 7).

Long-term use of inorganic fertilizer caused the accumulation of salt in topsoil (Chang et al., 2013). High soil salinity harmed most crops via the high osmotic stress, nutrient deficiencies, toxicity, and poor physical soil conditions (Chen et al., 2016). The previous study by Zhai, Yang & Hou (2015) found that salt stress inhibited the growth of tomato fruit, thereby decreasing the weight of single fruit and the yield from a single plant. However, some studies revealed that slight salt stress improved the tomato quality and increased the concentration of nutrient in the fruit (Hou & Shao, 2016; Wan et al., 2007). Even so, for those greenhouse soils subjected to strong evaporation and less water supply, the soil salinity was expected to be lower. In our study, a greater amount of irrigation water noticeably reduced soil salinity in plough layer, and promoted the movement of salt into the deeper soil layer, this confirmed the result by Zhang & Ai (2014). The close relationship between the soil salt content and irrigation quota also indirectly demonstrated that the salts in the experimental field were generally soluble. However, it should be noticed that the drip irrigation results in saturated pond beneath the dripper and then a zone of wetted soil with differing water content away from this pond extending both radially and vertically. The movement of soil moisture will influence the soil salinity. Hence the salinity profiles in our study could only reflect the status on the measuring date. More mechanism researches needs to be carried out in further research.

Various statistic models have been employed for the optimization of agricultural management strategies. Shao demonstrated the principal component analysis (Shao et al., 2014) and the entropy weight coefficient model (Shao et al., 2012) to be feasible in optimizing the subsurface drainage layout schemes for waterlogged field by considering crop yield, quality and water use. Similarly, Yan (2014) used entropy weight coefficient model to select the best water-fertilizer scheme for flue-cured tobacco, and the result given by the model has proved to be dependable. Another model, projection pursuit, was adopted by Hou & Shao (2016) to choose suitable irrigation and drainage scheme for the tomato in coastal saline field. In our study, the yield increase, water saving, as well as salinity reduction effects under the different schemes were considered, and the entropy weight coefficient model was used to optimize the coupling schemes of drip irrigation and organic fertilization by assessing their comprehensive benefits. In future, more statistic models could be applied to assess the agricultural practices.

In this study, we investigated the tomato yield, quality, water use and soil salinity under different water-fertilizer coupled treatments. Since bioorganic fertilizer has profound effects on soil microflora, the relationship between soil moisture and soil microbial activity (Cook & Orchard, 2008) might be an important research point to study the coupling effects of drip irrigation and bioorganic fertilizer application.

## Conclusion

The results demonstrated that the tomato marketable yield was significantly (*p* < 0.01) affected by irrigation or fertilization. The SAR of tomato were significantly (*p* < 0.05) affected by irrigation or/and fertilization. Fertilization had highly significant (*p* < 0.01) effect on the contents of soil nutrients (N, P, K), while the coupling effect of irrigation and fertilization was not pronounced. According to the multi-index analysis and the computed result by the entropy weight coefficient model, irrigation quota of 180 m^3^/ha in combination with organic fertilizer application amount of 4,400 kg/ha was the optimal water-fertilizer coupling strategy with the most satisfactory comprehensive benefits. The marketable yield, SAR and IWUE under this optimal strategy were 122.4 t/ha, 9.2, 32.4 kg/m^3^, respectively, and by 28.0%, 29.6% and 28.1% higher compared to that under CK.

##  Supplemental Information

10.7717/peerj.3855/supp-1 Data S1 Raw data

## Additional Information and Declarations

### Field Study Permissions

The following information was supplied relating to field study approvals (i.e., approving body and any reference numbers): Field experiments were approved by the Fruit Science and Technology Demonstrative Base of Yunxiao county.

### Data Availability

The following information was supplied regarding data availability: The raw data has been provided as Data S1.
